# Serum levels of interleukin-23 and 35 in patients with and without type 2 diabetes mellitus and chronic periodontitis

**DOI:** 10.22088/cjim.10.3.295

**Published:** 2019

**Authors:** Avideh Maboudi, Aida Eghbalian-Nouzanizadeh, Hajar Seifi, Adele Bahar, Mohadese Mohadese, Reza Ali Mohammadpour, Saeid Abediankenari, Seyedeh Leila Poorbaghi, Masood Sepehrimanesh

**Affiliations:** 1 1.Department of Periodontics, Diabetes Research Center, Faculty of Dentistry, Mazandaran University of Medical Sciences, Sari, Iran; 2 Student Research Committee, Faculty of Dentistry, Mazandaran University of Medical Sciences, Sari, Iran; 3 Department of Internal Medicine, Diabetes Research Center, Faculty of Medicine, Mazandaran University of Medical Sciences, Sari, Iran; 4 Dental Implant Research Center, Dentistry Research Institute, Tehran University of Medical Sciences, Tehran, Iran; 5 Biostatistics Department, Health Faculty, Diabetes Research Center, Mazandaran University of Medical Sciences, Sari, Iran; 6 6.Immunogenetics Research Center, School of Medicine, Mazandaran University of Medical Sciences, Sari, Iran; 7 7.Obesity Prevention and Treatment Research Center, Shiraz University of Medical Sciences, Shiraz, Iran; 8 New Iberia Research Center, University of Louisiana at Lafayette, New Iberia, Lousiana, USA

**Keywords:** Type 2 diabetes mellitus, Chronic periodontitis, Interleukin-23, Interleukin -35

## Abstract

**Background:**

Type 2 diabetes mellitus (DM) and chronic periodontitis (CP) show common pathophysiological features. We investigated the serum levels of IL-23 and IL-35 in people with type 2 DM and CP.

**Methods:**

In a cross-sectional study, 72 patients were divided into four equal groups: group A, participants without type 2 DM and CP; group B, patients with type 2 DM without CP; group C, patients with CP and without type 2 DM; and group D, patients with type 2 DM and CP. Demographic data were obtained and periodontal conditions including clinical attachment loss, bleeding on probing, plaque index, gingival index, and probing depth was evaluated on all existing teeth. Fasting blood sugar (FBS) levels, hemoglobin (Hb) A1c, erythrocyte sedimentation rate (ESR) and C-reactive protein (CRP) were assessed. In addition, serum levels of IL-23 and 35 were measured using enzyme-linked immunosorbent assay.

**Results:**

The serum levels of IL-23 and 35 showed no significant differences between all groups (P>0.05). A significant positive correlation between the serum concentration of IL-23 and clinical attachment loss in the control group (r: 0.548, P=0.019) was detected. A significant negative correlation between IL-35 and the plaque index in group B (r: -0.578, P=0.012), plus significant negative correlations between IL-23 with ESR (r: -0.487, P=0.040) and CRP (r: -0.498, P=0.035) in groups C and D were also detected.

**Conclusion:**

Despite significant associations of serum concentration of IL-23 and 35 with certain periodontal and inflammatory indices, neither type 2 DM nor CP differentially affects serum levels of these two cytokines.

Periodontitis is an inflammatory condition of the teeth-protecting tissues that is induced mostly by gram-negative and anaerobic species microorganisms (1, 2). Periodontitis results in progressive degeneration of periodontal ligaments, alveolar bone, gingiva, and eventual loss of teeth (3). Gingival plaques contain living bacteria and their biofilm bacteria products such as lipopolysaccharides (LPS) that induce inflammation. The inflamed tissues then release inflammatory cytokines into the bloodstream as signaling proteins (4). On the other hand, diabetes mellitus (DM) is known as a significant risk factor for periodontitis (5, 6). Persistent hyperglycemia, which is seen in prediabetes and diabetic patients, induces damages in the periodontal tissues due to the changes in the activity of polymorphonuclear cells and leukocytes plus alteration of glycosaminoglycans and cytokines production (7). 

Thus, patients with type 2 DM have a high prevalence of gingivitis, periodontitis, oral candidiasis, and xerostomia (dry mouth). The severity of these diseases depends on the duration of the patient's diabetes (8).

Interleukin (IL)-23, which is a signaling protein in inflammatory conditions of periodontitis, also plays a role in mild degrees of type 2 DM-related inflammation (9). In chronic infections, antigens stimulate dendritic cells, macrophages, and IL-23 production that motivates the production of IL-17. Also, IL-23 increases the production of IL-6, IL-1 and tumor necrosing factor (TNF)-α in the autocrine/paracrine pathway (10). Moreover, IL-35 is an anti-inflammatory cytokine in the IL-12 family that plays a suppressive role in the immune system (11). Since IL-23 is involved in the pathogenesis of type 2 DM and chronic periodontitis (CP), and there are controversies in some studies (12, 13) and lack of any data about the role of IL-35, we aimed to investigate the serum levels of IL-23 and IL-35 in people with type 2 DM and CP and compare them with the control group.

## Methods

**Patients: **The current cross-sectional study was done in 2016 with the coordination of three private and public dental centers of Sari, North of Iran. Based on the prevalence of CP and DM and the following formula, α=0.05 was considered significant, and the beta level was set at β=0.10. Additionally, four study groups and a sample size of 18 patients per group were calculated for this preliminary study. Based on convenience sampling method, referred patients were followed-up to identify those with DM based on the criteria of the American Association of Diabetes (14) and patients with CP based on the existence of inflammation in the periodontium (15). 

**Figure 1:**
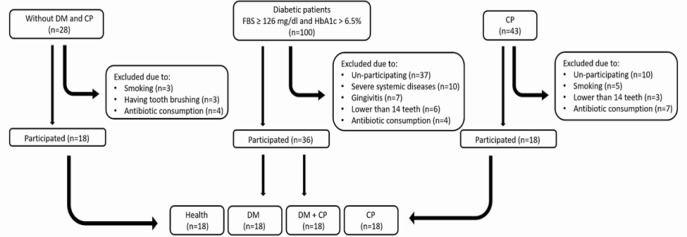
Flow chart of patients’ enrollment. DM, diabetes mellitus; CP, chronic periodontitis

Briefly, type 2 DM was confirmed if one of the following conditions was reported: (a) fasting blood sugar (FBS) ≥ 126 mg/dL; (b) random blood sugar ≥ 200 mg/dL with signs of hyperglycemia; and (c) HbA1c ≥ 6.5%. Periodontitis was also diagnosed in patients with at least one site with ≥ 3 mm probing depth and ≥ 2 mm loss of clinical attachments. Exclusion criteria were: patients younger than 18 year, progressed renal failure with serum creatinine level > 2 mg/dL, class III and IV heart failure, hepatic failure, use of certain drugs that induce insulin resistance and hyperglycemia such as prednisolone, cyclosporine and etc., consumption of antibiotics during the past three months, being treated for periodontitis, smoking during pregnancy, hemorrhagic diseases, and those who had less than 14 teeth (Fig. 1). Four groups, each with 18 patients were defined as follows:

Group A: participants with neither type 2 DM nor CP

Group B: patients with type 2 DM based on laboratory test and without CP based on clinical examination

Group C: patients with CP and without type 2 DM

Group D: patients with type 2 DM and CP

The Ethics Committee of Mazandaran University of Medical Sciences approved the protocol of the study (No. 13959); hence, all investigations have been carried out in accordance with the principles of the Declaration of Helsinki as revised in 2008. Moreover, the aims of the study were described completely for all enrolled participants and written consent informs were obtained.

**Evaluations:** A face-to-face questionnaire about demographic data, familial history of type 2 DM, and education level was filled out. Then, the periodontal examination was performed using a mirror and a periodontal probe (Williams probe, Medisporex, Pakistan). Clinical attachment loss (CAL) (16), bleeding on probing (BOP) (17), plaque (Loe) index (PI) (18), gingival index (GI) (19) and probing depth (PD) (20) for all teeth were recorded by an expert dentist (the first author). Her correlation coefficient of measurements was 0.84 in the previous pilot study using five CP patients and two measurements with a one-week interval.

**Serum measurements:** About 4 ml of fasting blood samples obtained from the cubital vein into sterile vacutainers without anticoagulant and serum were harvested after centrifugation (3000 RPM, 10 min). Serums were used for measurements of FBS (mg/dL) using an enzymatic colorimetric method (GOD/PAP) by an autoanalyzer prestige 24i (Tokyo Boeki Medical System, Japan), C-reactive protein (CRP, mg/l) by the nephelometric method, IL-23 and IL-35 concentrations (pg/ml) using the sandwich ELISA (Ebioscience, San Diego, CA, USA). Another 2 ml of the previously drawn blood was placed into the EDTA tube used for measuring HbA1c (%) using the Nycocard Reader II and erythrocyte sedimentation rate (ESR) (mm/h) by the manual method (21, 22).

Our analysis variables had a biological nature and a normal distribution in the population. In addition, we checked the data by the Kolmogorov-Simonov test, and find, with the exception of one or two data that was far from average, all other data had a normal distribution. Therefore, we handled and analyzed them using parametric analyses. All statistical analyses were performed using the software SPSS Version 23. Comparison of age, serum, blood biomarkers, and periodontal indices between the four groups was performed using the one-way analysis of variance (ANOVA) and Tukey's Post Hoc test. Associations between jobs, education, systemic diseases, familial history of type 2 DM, gender, severity, and distribution of periodontal diseases with type 2 DM and CP were checked using the chi-square test. Associations between serum concentrations of all quantitative variables were checked using the Pearson’s correlation test. A p<0.05 was considered as the significant difference. 

## Results

Subjects in the healthy control group had a significantly lower age (P<0.001) and a higher number of teeth (P<0.05) in comparison with the other three groups. Since the enrollment criteria for the study patients did not include their sex, our four groups were not sex-matched (P<0.001); hence, we had a higher frequency of females in the all groups except the CP group. Most of the participants were unemployed or homemakers (44.4%) while the others had a diploma or associate degree (38.9%) and were without systemic diseases (73.6%) or familial history of DM (52.8%). 

Generalized moderate periodontitis was the most frequent form of periodontal diseases in both CP and DM+CP groups (66.7% and 44.4%, respectively) followed by severe diffused periodontitis (16.7% and 38.9% respectively, table 1). FBS and HbA1c were significantly higher in the DM and DM+CP groups in comparison with the other two groups, but no significant differences were detected between these two groups with regard to FBS or HbA1c levels.As shown, all periodontal indices in the CP and DM+CP groups were significantly higher than related indices in the control and DM groups (P<0.05). Despite the significantly lower GI index in the DM+CP group compared with the CP group (P=0.03), no significant differences were detected between the control and DM groups, or the CP and DM+CP groups (p>0.05).

**Figure 2:**
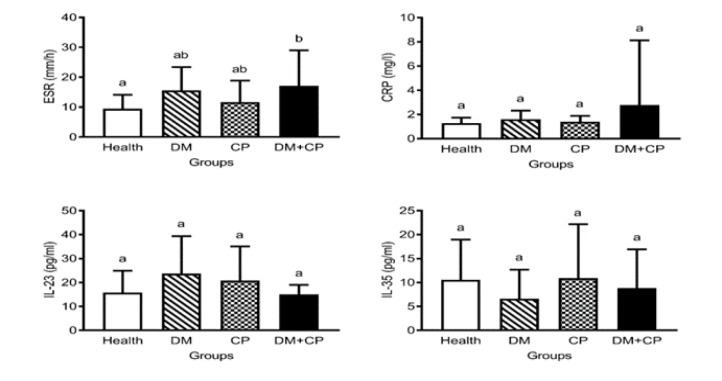
Comparison of serum biochemical indices of diabetes and inflammation between the four groups. DM, diabetes mellitus; CP, chronic periodontitis. Significant statistical differences between groups were indicated by different superscript letters (P<0.05)

Accordingly, fig. 2 shows that the DM+CP showed higher inflammation in comparison to the control group, which was based only on higher ESR. However, CRP, IL-23, and IL-35 levels showed no significant differences between the four groups. As a secondary finding, comparisons of periodontal indices of the mandible and maxilla were performed separately. 

Our findings were similar to the total indices that have been presented above, except for the lack of significant difference in the PD of the maxillary teeth between the DM+CP and the other three groups and in the GI of the mandible and maxilla between the DM+CP and CP group (p<0.05, data not shown). 

Significant negative correlations were seen between IL-35 with PI in DM group; IL-23 with HbA1c and ESR in CP group and between IL-23 with CRP in DM+CP group. Moreover, significant positive correlations were detected between IL-23 with age and CAL in the control group and between IL-23 and PI in the CP group.

**Table 1:** Demographic characteristics of patients in four groups

	**Groups**				
**Variables**	**Health (n=18)**	**DM (n=18)**	**CP (n=18)**	**DM+CP (n=18)**	**Total**
Age (years)	27.44±10.07^a^	46.06±10.53^b^	44.78±13.18^b^	52.72±9.96^b^	
Number of tooth	29.00±2.59^a^	24.50±5.37^b^	24.94±4.14^b^	24.33±4.70^b^	
Gender					
Male	5 (27.8)	3 (16.7)	14 (77.8)	3 (16.7)	25 (34.7)
Female	13 (72.2)	15 (83.3)	4 (22.2)	15 (83.3)	47 (65.3)
HbA1c	5.51±0.36^a^	8.53±1.00^b^	5.79±0.45^a^	8.12±1.09^b^	
FBS	86.56±5.23^a^	184.00±68.91^b^	89.44±8.91^a^	154.39±43.55^b^	
Periodontal indices					
Upper tooth					
CAL	0.72±2.27^a^	3.72±6.26^a^	47.61±31.35^b^	46.83±29.97^b^	
BOP	8.17±6.53^a^	15.17±11.62^a^	33.33±17.16^b^	31.55±13.98^b^	
PI	25.06±23.63^a^	47.17±22.26^a^	81.67±37.11^b^	72.94±19.42^b^	
GI	32.11±26.43^a^	43.67±29.10^a^	102.44±44.32^b^	74.50±27.44^b^	
PD	89.11±21.71^a^	89.94±23.87^a^	118.83±33.06^b^	111.17±26.59^ab^	
Lower tooth					
CAL	3.11±5.78^a^	4.39±5.65^a^	64.39±35.30^b^	92.61±55.30^b^	
BOP	5.61±4.50^a^	12.11±9.85^a^	38.89±15.49^b^	32.44±15.93^b^	
PI	28.78±22.67^a^	41.72±17.16^a^	95.67±33.39^b^	88.94±25.46^b^	
GI	28.44±25.58^a^	39.50±26.95^a^	110.00±28.20^b^	83.78±31.67^b^	
PD	85.22±13.17^a^	77.00±28.53^a^	124.50±25.15^b^	113.50±33.55^b^	
Jobs					
UE or housekeeper	0 (0)	11 (61.1)	7 (38.9)	14 (77.8)	32 (44.4)
Self-employment	3 (16.7)	4 (22.2)	8 (44.4)	3 (16.7)	18 (25.0)
Employee	14 (77.8)	2 (11.1)	3 (16.7)	1 (5.6)	20 (27.8)
Retired	1 (5.6)	1 (5.6)	0 (0)	0 (0)	2 (2.8)
Education					
Before the diploma	0 (0)	8 (44.4)	8 (44.4)	10 (55.6)	26 (36.1)
Diploma or AD	4 (22.2)	8 (44.4)	8 (44.4)	8 (44.4)	28 (38.9)
BSc and higher	14 (77.8)	2 (11.1)	2 (11.1)	0 (0)	18 (25.0)
Systemic diseases					
No	18 (100)	10 (55.6)	16 (88.9)	9 (50.0)	53 (73.6)
Hypertension	0 (0)	2 (11.1)	0 (0)	6 (33.3)	8 (11.1)
Thyroid disorders	0 (0)	3 (16.7)	0 (0)	1 (5.6)	4 (5.6)
CVDs	0 (0)	0 (0)	1 (5.6)	1 (5.6)	2 (2.8)
Others	0 (0)	3 (16.7)	1 (5.6)	1 (5.6)	5 (6.9)
Familial history of DM					
Yes	1 (5.6)	16 (88.9)	2 (11.1)	15 (83.3)	34 (47.2)
No	17 (94.4)	2 (11.1)	16 (88.9)	3 (16.7)	38 (52.8)
Periodontitis					
No	18 (100)	18 (100)	0 (0)	0 (0)	36 (50.0)
Localized mild	0 (0)	0 (0)	0 (0)	0 (0)	0 (0)
Generalized mild	0 (0)	0 (0)	2 (11.1)	3 (16.7)	5 (6.9)
Localize moderate	0 (0)	0 (0)	1 (5.6)	0 (0)	1 (1.4)
Generalized moderate	0 (0)	0 (0)	12 (66.7)	8 (44.4)	20 (27.8)
Localized severe	0 (0)	0 (0)	0 (0)	0 (0)	0 (0)
Generalized severe	0 (0)	0 (0)	3 (16.7)	7 (38.9)	10 (13.9)

UE, un-employed; AD, associate degree; CVDs, cardiovascular diseases; CAL, clinical attachment loss; BOP, bleeding on probing; PI, plaque (Loe) index; GI, gingival index; PD, probing depth. Different superscript letters in each row indicate statistical significant difference (P<0.05).

## Discussion

In the preset study, patients with DM+CP showed higher inflammation (ESR index) in comparison to the control group while CRP, IL-23, and IL-35 levels were similar between the four groups.

Periodontal disease is the sixth most common complication of DM (23), and these two diseases have common pathophysiological mechanisms that have been confirmed by animal (24) and human studies (25). Also, patients with uncontrolled DM show a higher prevalence of periodontal diseases (26). It has been reported that DM-induced over-expression of IL-17 as a pro-inflammatory cytokine (27) in addition to its stimulated expression by IL-23 (28). These two cytokines along with other inflammatory molecules and cells play important roles in the common pathological pathway of DM and CP (29). However, we found no significant difference in the serum concentration of IL-23 between our four groups. 

Similar to our findings, Vieira Ribeiro et al., by evaluating different cytokines in healthy patients with DM and chronic periodontitis reported that no significant difference in the level of IL-23 among healthy and controlled diabetic patients (13). Furthermore, Duarte et al. reported that CP- not DM- significantly altered the expression of the immune system and inflammatory markers related genes such as IL-23 (30), which is similar to our report about DM but opposite regarding CP. Moreover, santos et al.detected significant differences in the gingival crevicular fluid (GCF) level of IL-4 and IL-17, not IL-23, between well-controlled and poorly controlled DM subjects with CP before and after periodontal therapy (12). Changes in the IL-17 without any changes in the IL-23 may be due to the regulatory roles of other cytokines involved in the stimulation of IL-17which affect the proliferation and differentiation of Th17 as the producers of IL-17. On the other hand, there may be differences in the source of the biomarker used in the different studies, which could potentially cause the controversial findings. For instance, the inflammatory role of IL-23 was reported by assessing this biomarker in the gingival biopsy (31) and GCF (32) of patients with CP. However, when we assessed the serum level of IL-23, we did not confirm such role. 

Significant negative correlations between IL-23 with ESR and CRP in the CP and DM+CP groups were seen, respectively, which seems irrational due to the inflammatory nature of all mentioned parameters. Significant positive correlation between the serum concentration of IL-23 and CAL in the control group was also reported previously in gingivitis and periodontitis tissues (33). In addition, novel significant positive correlation between serum IL-23 concentration and GI is rational and could be investigated further in future research. There are no reports about the comparison of serum levels of IL-35 between patients with and without DM and CP. In addition, no studies have evaluated IL-35 and IL-23 concurrently in DM. Only one study evaluated the expression of IL-23 and IL-35 in GCF and serum of CP and compared their levels with healthy volunteers. They found that the GCF and serum levels of both cytokines were elevated in CP patients (34). Moreover, an increase in the serum level of IL-35 in patients with both types of DM (35) and periodontal diseases (36) were reported separately. Due to the immunosuppressive role of IL-35 and its selective activities to proliferate regulatory T cells and suppress Th17 cells (37), the negative associations between IL-23 and IL-35 is conceivable. However, such a relationship was not detected in our study, which may be related to the evaluation of both biomarkers in the serum - not GCF or gingival biopsy. Contrary to those reported by Koseoglu et al. in the GCF of patients with chronic periodontitis (38), the significant negative correlation between IL-35 and PI in DM group was found in this study, which is also rational based on the anti-inflammatory properties of this immunosuppressive cytokine. This result is about similar to the recent finding of Kaustubh et al. about significantly higher GCF IL-35 levels in a healthy population compared with patients with gingivitis (39). 

Other non-significant correlations between any periodontal indices and serum concentrations of IL-35 could reinforce this hypothesis that periodontitis and gingivitis induce no changes in the expression of IL-35. However, a recent study by Kaustubh et al. has reported the suppressing role of IL-35 in gingival inflammation and maintaining periodontal health (40). For more validation and confirmation, it is recommended to evaluate the concentration of IL-35 in GCF and saliva in healthy participants and patients with periodontitis and gingivitis.

Despite our findings, the low number of patients in each group was the one important limitation of our study. The limitation was due to our inclusion/exclusion criteria that limited the number of patients with diabetes and periodontitis with a minimum number of 14 teeth. 

In conclusion, no significant differences in the serum levels of IL-23 and IL-35 were detected between patients with and without DM and CP in this preliminary case-control study. It seems that the role of these two ILs should be further evaluated and confirmed in future research, especially cohort studies.
